# The quest for canine leishmaniasis in Romania: the presence of an autochthonous focus with subclinical infections in an area where disease occurred

**DOI:** 10.1186/s13071-016-1583-5

**Published:** 2016-05-21

**Authors:** Mirabela Oana Dumitrache, Yaarit Nachum-Biala, Matan Gilad, Viorica Mircean, Cristina Daniela Cazan, Andrei Daniel Mihalca, Gad Baneth

**Affiliations:** Department of Parasitology and Parasitic Diseases, Faculty of Veterinary Medicine, University of Agricultural Sciences and Veterinary Medicine Cluj-Napoca, Cluj-Napoca, Romania; Koret School of Veterinary Medicine, Hebrew University of Jerusalem, Rehovot, Israel

**Keywords:** *Leishmania infantum*, Romania, Canine leishmaniasis, ELISA, PCR

## Abstract

**Background:**

Canine leishmaniasis is a severe, potentially life-threatening, systemic vector-borne disease of dogs caused by protozoan parasites of the genus *Leishmania*. Romania has been traditionally regarded as a non-endemic country for leishmaniasis with sporadic human disease cases. However, the recent report of an autochthonous canine leishmaniasis case (the first in the last 80 years) suggested the presence of an infection focus in the area of Râmnicu Vâlcea. The present study describes a survey of canine leishmaniasis in this geographical area with comparison to a georeferenced dataset of sand fly distribution based on historical literature records.

**Methods:**

The study was carried out in Râmnicu Vâlcea and included samples (serum, blood and conjunctival swabs) collected from 80 dogs including client-owned dogs from two local practices and dogs from two public shelters. Serum anti-leishmanial antibodies were assessed by ELISA. All blood and conjunctival samples were assessed by real-time quantitative PCR, targeting the leishmanial kinetoplast minicircle DNA.

**Results:**

Three dogs (3.7 %) were seropositive and another four (5.0 %) showed borderline results indicative of exposure or infection. TaqMan PCR was performed for all dogs, on both blood and conjunctival swabs. Seven dogs (8.7 %) were positive by conjunctival swab PCR and one dog (1.2 %) by blood PCR. None of the positive dogs presented clinical signs compatible with canine leishmaniasis.

**Conclusions:**

This is the first study evaluating canine leishmaniasis in a dog population in Romania by both highly sensitive PCR and serology. Although the prevalence was relatively low compared to other endemic regions, our results clearly demonstrate the presence of a canine leishmaniasis focus in Romania.

**Electronic supplementary material:**

The online version of this article (doi:10.1186/s13071-016-1583-5) contains supplementary material, which is available to authorized users.

## Background

Canine leishmaniasis (CanL) is a severe zoonotic disease caused by protozoan parasites of the genus *Leishmania*. At least 12 species of *Leishmania* have been reported to infect dogs in the Old and New World. However, the most important etiological agent of CanL in Europe is *Leishmania infantum* that also causes visceral and cutaneous leishmaniasis in humans [1]. Zoonotic visceral leishmaniasis is considered the most widespread form of zoonotic leishmaniasis, and if left untreated, can be fatal [2]. Dogs are the main peridomestic reservoirs of *L. infantum* whereas jackals, wolves and foxes are sylvatic hosts. Phlebotomine sand flies are the biological vectors of all forms of leishmaniasis [2, 3].

CanL is endemic in more than 70 countries in Europe, Africa, Asia and the Americas, occurring mainly in the Mediterranean region and South America. It is often diagnosed in non-endemic countries, where imported cases or sporadic autochthonous cases are increasingly posing a veterinary and public health concern and there is therefore an imperative need for epidemiological studies to investigate the prevalence and spread of infection [3, 4]. Although it is hard to assess whether there is a real or an artificial increase of the global incidence of leishmaniasis (due to increased awareness and improved reporting), it is widely accepted that CanL is a dynamically expanding complex zoonosis, with continuously changing transmission patterns [2]. It has been estimated that only in the western Mediterranean countries, at least 2.5 million dogs (16.7 %) are infected [5] and there is an evident northward expansion of CanL in Europe, as demonstrated in Spain [6, 7] and in Italy [8–10]. Autochthonous case reports in canids or epidemiological studies in countries from eastern Europe: Croatia [11], Bulgaria [12] and Hungary [13] suggest that the disease is spreading also eastwards.

Romania has been traditionally regarded as a country with sporadic cases of human leishmaniasis [14]. Since 1912, when the first case of autochthonous human leishmaniasis in Romania was described [15], 26 additional autochthonous cases have been reported (two isolated cases and one outbreak) [16–18]. The first report of clinical autochthonous CanL was published in 1934 [19]. These reported human and canine cases occurred in counties located in southern Romania (Prahova, Giurgiu and Dolj). Between 1969 and 2013, no autochthonous cases of *Leishmania* infection were reported, and human or canine cases diagnosed locally were all imported. This was regarded as a consequence of the widespread use of insecticides between 1958 and 1964, during the malaria eradication programmes [16].

In 2014, the first clinical case of autochthonous CanL within the last 80 years was reported by us, in a six year old mixed-breed bitch from Râmnicu Vâlcea (southern Romania). This dog had no history of travel abroad [20].

This study aimed to perform targeted surveillance with serological and molecular diagnostic techniques for CanL in the geographical area (Râmnicu Vâlcea) of this autochthonous CanL case. As no recent data on species composition and geographical distribution of the sand fly fauna and potential disease vectors in Romania are available, a review of the literature is also provided.

## Methods

### Study area, animals and sample collection

The study was carried out in Râmnicu Vâlcea (45.099672 N, 24.369317E) situated in the valley of the Olt River. The total population of this town was 118,887 in 2015; there are no available data or estimation regarding the dog population in Râmnicu Vâlcea.

The present study included samples collected in July-August 2014 from dogs that visited two local practices for general medical consultation, vaccination or external/internal parasitic treatment, and from dogs hosted in two local public shelters. All samples were collected with the consent of the owners or the shelter management. For all sampled dogs, general data (breed, sex, age, previous visits abroad) and clinical data compatible with CanL (skin and ocular lesions, lymphadenomegaly, weight loss and epistaxis) were registered. Conjunctival samples were obtained from both eyes using sterile bacteriology swabs without gel from all animals as previously described [21]. Blood was collected from all dogs by cephalic venipuncture in EDTA and clot tubes for serum. Clot tubes were centrifuged and serum samples were stored at -20 C° to be used for serology. Conjunctival swabs and blood samples in EDTA were preserved at -20 °C until processed for DNA extraction.

### ELISA serology and PCR

Serum anti-leishmanial antibodies were assessed by ELISA using crude leishmanial antigen as previously described [22]. All dog sera were diluted to 1:100, added in *L. infantum* antigen-coated plates and incubated for one hour at 37 °C. The plates were washed with 0.1 % Tween 20 in 50 nM phosphate-buffered saline (PBS) and incubated with Protein A conjugated to horseradish peroxidase for one hour at 37 °C. The plates were developed by addition of the substrate 2, 2-azino-di-3-ethylbenzthiazolihne sufonate (ABTS) (Boehringer Mannheim, Germany). Each plate was read at a wavelength of 405 nm, after the absorbance of the positive control canine reference serum reached a value between 0.95 and 1.0. In order to monitor inter-assay variation, positive and negative control dog sera were included on each plate. Optical density of each sample was calibrated against a positive control. A sample was considered positive if the calibrated optical density was above 0.6, as previously reported [23], while samples with calibrated optical density ranging between 0.2 and 0.6 were considered as borderline (BL).

Genomic DNA was extracted from all blood samples using a commercial kit (Isolate II Genomic DNA Kit, Bioline, London, UK) according to the manufacturer’s instructions. DNA was extracted from one conjunctival swab for each dog using the phenol-chloroform-isoamyl alcohol method as previously described [21]. All samples were assessed by real-time quantitative PCR, targeting the kinetoplast minicircle DNA from *L. infantum*, using TaqMan-MGB probe and PCR primers LEISH-1 and LEISH-2, according to Francino et al. [24].

### Statistical analysis and mapping

Frequency, prevalence and its 95 % confidence intervals (95 % CI), were calculated. Chi-square test was used for evaluating statistical significance; a *P*-value < 0.05 was considered statistically significant. Analysis was performed using the EpiInfo^TM^ 2000 (CDC, USA, http://www.cdc.gov/epiinfo/index.html) software. Cohen’s kappa coefficient was calculated in order to evaluate the inter-rater agreement between diagnostic methods, using the Win Episcope 2.0 software [25].

In order to review the distribution of the sand fly species in Romania and *Leishmania* spp. reports in dogs, a comprehensive literature survey (using Google Scholar and national library systems databases) has been performed and distribution data was georeferenced based on locality names, using Google Maps. Distribution maps were generated using the QGIS software (http://www.qgis.org).

## Results

### Detection of the infection in dogs

A total number of 80 dogs were tested for *L. infantum* infection. None of these showed clinical signs characteristic for CanL. The distribution of dogs by origin and the results of the serological and molecular diagnostic techniques are shown in Table 1. Three dogs (3.7 %) were seropositive, while an additional four (5.0 %) showed BL results. All seropositive dogs originated from the same clinical practice. When both seropositive and BL samples were considered, 7 dogs (8.7 %) were reactive with *L. infantum* antigen. TaqMan PCR was performed for all dogs, on both blood and conjunctival swabs. Seven dogs (8.7 %) were positive by conjunctival swab PCR and one dog (1.2 %) by blood PCR. The cumulative positivity for each dog is shown in Table 2. A moderate agreement (*k* = 0.58) was found between ELISA serology and conjunctival swab PCR.

**Table 1:** The number of dogs surveyed for leishmaniasis in each collection site and frequency of positive or borderline results by ELISA and positivity by PCR

Dog provenience	Number of dogs sampled	Serology		PCR	
		No. of positive (% of total no. tested)	No. of borderline (% of total no. tested)	No. of positive (blood) (% of total no. tested)	No. of positive (swab) (% of total no. tested)
Shelter 1	30	0 (0)	3 (10)	0 (0)	1 (3.3)
Shelter 2	16	0 (0)	1 (6.2)	1 (6.2)	1 (6.2)
Practice 1	8	0 (0)	0 (0)	0 (0)	0 (0)
Practice 2	26	3 (11.5)	0 (0)	0 (0)	5 (19.2)
Total	80	3 (3.7)	4 (5)	1 (1.2)	7 (8.7)

**Table 2:** The cumulative positivity for different diagnostic tests

Sample ID	Origin	Breed	Age (years)	Positive serology	Borderline serology	PCR Positive (blood)	PCR Positive (swab)
14	Shelter 1	Mixed-breed	1.5	No	Yes	No	No
16	Shelter 1	Mixed-breed	8	No	Yes	No	No
24	Shelter 1	Mixed-breed	2	No	Yes	No	No
25	Shelter 1	Mixed-breed	5	No	No	No	Yes
39	Shelter 2	Mixed-breed	5	No	No	No	Yes
50	Shelter 2	Mixed-breed	1	No	No	Yes	No
53	Shelter 2	Mixed-breed	15	No	Yes	No	No
60	Practice 2	Chihuahua	8	Yes	No	No	Yes
63	Practice 2	Bichon	9	No	No	No	Yes
68	Practice 2	Poodle	10	Yes	No	No	Yes
72	Practice 2	Dachshund	6	Yes	No	No	Yes
79	Practice 2	Transylvanian Scenthound	5	No	No	No	Yes

None of the dogs sampled in the clinical practice has travelled abroad and the parents of the owned dogs did not have a travel abroad history nor were the dogs reported to mate with dogs travelling to endemic countries. For the dogs in the public shelters, this information was not available, but as these are stray dogs with no owners, it is highly improbable that they had a long-distance travel history. There were no statistically significant differences in the seroprevalence and molecular prevalence between different dog breeds, sexes or age.

### Review of the sand fly distribution and historical canine leishmaniasis reports in Romania

**Fig. 1:**
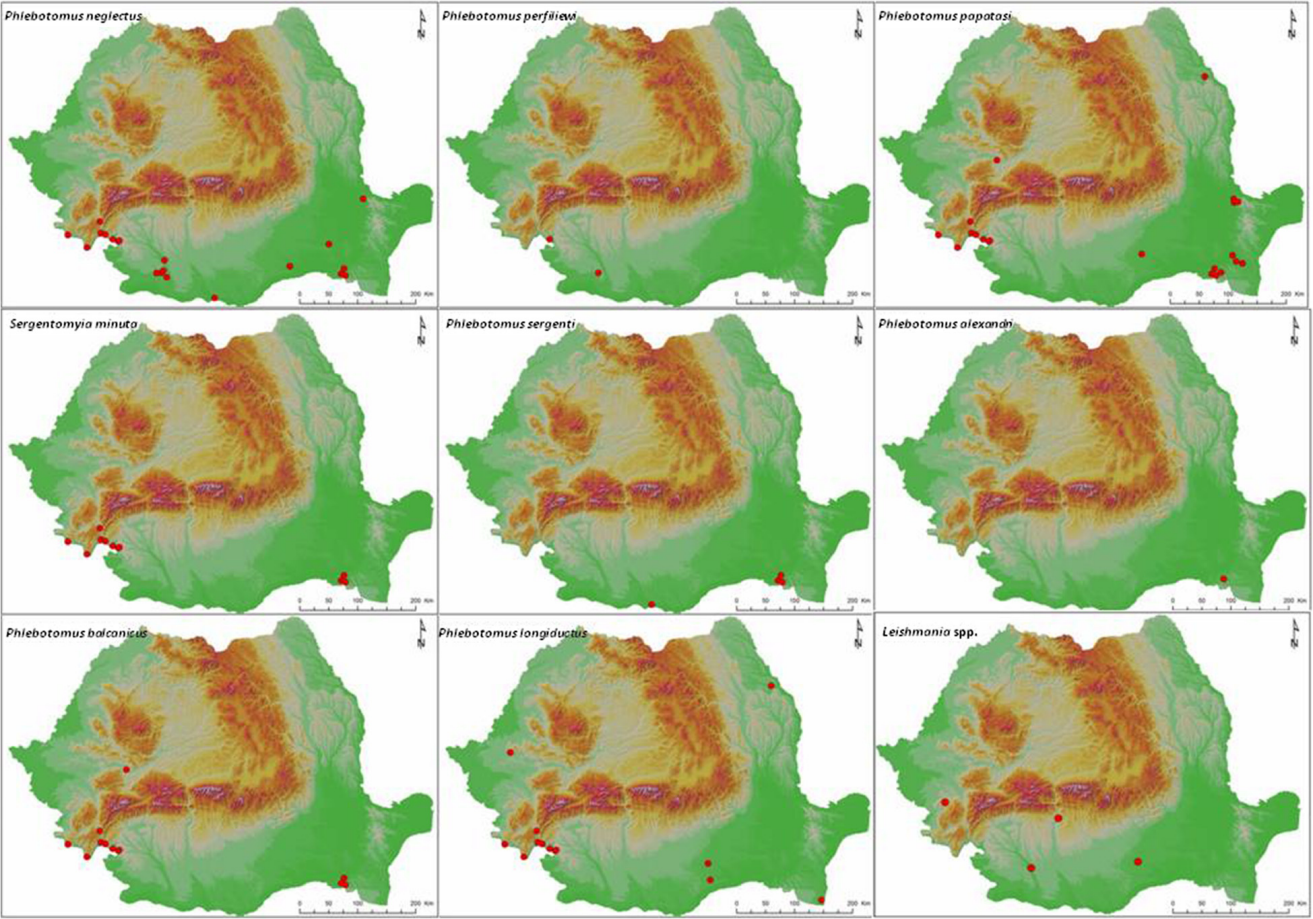
Geographical distribution of sand fly species in Romania and *Leishmania* spp. reports in dogs

In total, 84 georeferenced records were extracted for sand fly distribution and four for CanL (Additional file 1: Table S1) [26–31]. This offers a complete dataset of geographical coordinates and distribution maps for each one of the eight reported sandflies species and historical CanL reports (Fig. 1).

## Discussion

The recent occurrence of a clinical case of autochthonous CanL in Romania after 80 years since the previous reports [20] raised questions about the presumed non-endemic epidemiological status of this zoonotic disease in the country and highlighted the need for both targeted epidemiological studies and extensive, long-term epidemiological surveys.

This study performed in the same location of the case report, showed a higher molecular prevalence than seroprevalence, as in various other studies in endemic areas [32, 33]. The difference between PCR and serology can be explained by the fact that in endemic areas, only a part of the infected dogs which are PCR-positive develop anti-*Leishmania* antibodies [3, 34]. Furthermore, the conjunctival swabs have been found to be more suitable for the detection of *Leishmania* spp. DNA than blood, [35] explaining the higher prevalence of conjunctival-positive dogs in this study, compared to blood-positivity in a single dog.

None of the positive dogs presented clinical signs compatible with CanL, regardless of the detection method. These results are in agreement with CanL surveys from other countries where the majority of infected dogs were affected sub-clinically and did not exhibit clinical signs. In a study performed in Mallorca, Spain, 15 out of 26 seropositive dogs were clinically healthy [34]. Fakhar el al. [36] showed that 88 % (22/25) of the PCR-positive and 67 % (4/6) of the seropositive dogs were asymptomatic. Since the majority of positive dogs exhibit no clinical signs, it is likely that infected, but asymptomatic dogs play an active role in the transmission of *Leishmania* spp., acting as sources of infection for phlebotomine vectors [34, 37]. Moreover, Guarga et al. [38] showed that sandflies are more attracted by healthy skin of infected dogs and the interest of the female sand fly for feeding will decrease as the disease progresses and more skin areas become severely inflamed.

Four of the investigated dogs presented borderline results. These animals are indicative of exposure to *L. infantum* and are likely to be infected and progressing towards seropositivity. Long-term studies have shown that infected dogs can follow different patterns of progression [3]. Some dogs will develop clinical signs of disease shortly after infection, whereas others will remain infected for a long period of time, without exhibiting lesions and clinical disease. However, this might not be a permanent condition and any change in the health status of these dogs could lead to the activation of the infection and the development of clinical disease [3].

Only a few studies were performed in order to evaluate the prevalence of CanL in Romania. Following an outbreak of human leishmaniasis involving 23 children and one adult in 1954 [18], two studies were carried out in the human focus area in order to identify animal reservoirs. In Dolj County, the microscopical prevalence in bone marrow aspirates of CanL was 1.2 % [39], while in the neighbouring county of Caraș-Severin, the prevalence was 2.2 % [40]. All positive dogs were infected sub-clinically. In a more recent study of dogs from the Bucharest area, out of 138 sera tested by IFAT, four (2.9 %) were found positive for anti-*Leishmania* spp. antibodies. All animals were apparently clinically healthy [41].

An interesting finding in our study is that all seropositive dogs and five out of seven dogs positive by conjunctival swab PCR had visited the same clinical practice (Practice 2). This clinic provides service in the same neighbourhood, along the river, in which the clinical case by Mircean et al. [20] was found. This may be suggestive for the presence of a disease focus.

It is not clear if the focus of infection identified in the present study has been present for a long time, as the disease status has never been investigated and infection seems to be mostly subclinical, or if it represents an emerging new focus. The situation is particularly interesting, as Romania is located at the northern border of sand fly distribution in Europe, like several other countries where the epidemiology of CanL has been assessed in the past. In a three-year serological survey performed at a public kennel in the Bologna province in Northern Italy, a low endemic region, the prevalence of infected dogs ranged between 4.9 and 12.6 % showing the presence of an active focus of infection [10]. Until recently, the north of Spain has been considered a non-endemic area. However, after examining a total of 418 stray dogs for the infection with *L. infantum*, an overall seroprevalence of 3 % was detected on the Cantabrian coast [6]. In a recent epidemiological study assessing infection with different vector-borne pathogens in dogs from Romania and Hungary, the prevalence of anti-*Leishmania* antibodies was 2.9 % in dogs from Romania and a molecular prevalence of 2.6 % was registered in dogs from Hungary by real-time PCR [41].

Over the past few years, different epidemiological studies or isolated case reports highlighted an increase in the global incidence of CanL and a clear northward and eastward expansion [7, 10, 13, 20]. Although the factors generating such phenomenon are not easy to identify, it seems that climate change and demographic factors are playing a major role in this evident disease expansion. Particularly, in temperate zones, climate change could affect the distribution of leishmaniasis by the effect of increased average temperatures on existing phlebotomine species or the establishment of tropical and/or subtropical species [2].

Additionally, the role of wildlife reservoirs cannot be excluded, as they are known to be important factors in disease transmission. The role of wildlife in *Leishmania* spp. transmission has not been assessed yet in Romania. However, Romania has considerably dense wild canid populations including wolves, foxes and the recently territory-expanding golden jackals [42].

No recent data regarding species composition and geographical distribution of the sand fly fauna in Romania are available. However, a review of some older publications reports the presence of eight sand fly species in Romania [16], but without reporting geographical locations. Two of the species reported in Romania, *Phlebotomus perfiliewi* and *P. neglectus* are known as competent vectors for *L. infantum* [43].

Despite the recent autochtounous human and CanL reports, leishmaniasis is considered absent in Romania by most of the practicing veterinarians and physicians. Moreover, the awareness of the public regarding the zoonotic character of the disease and its association with dogs is relatively low, and no proper prevention of sand fly bites is performed routinely in dogs. Therefore, it is imperative that the presence of a CanL focus described in this study, as well as the possibility that other foci may exist, will be communicated to veterinary and public health officials in order to raise the awareness to this potentially fatal zoonosis.

## Conclusions

This is the first study to evaluate the prevalence of CanL in Romania by sensitive PCR and serology, in an area from which a recent clinical report of autochthonous infection was made. Although the prevalence is relatively low compared to some other endemic regions, our results clearly demonstrate the presence of a CanL focus in Romania, a country at the border of the disease distribution. Further studies already initiated under the frame of the VectorNet project managed by EFSA and the ECDC, will investigate the presence and abundance of the sand fly vectors in the area. Furthermore, awareness of public health and veterinary professionals to the possibility of clinical leishmaniasis in dogs and humans should be increased in Romania.

### Ethical approval and consent to participate

All samples were collected and used with the owner consent and collected by professional vets in the clinical facilities. As the studies did not involve any experimental work, no ethical committee approval was required.

## Additional file

Additional file 1:
**Table S1.** Geographical distribution of sand fly fauna in Romania. (XLSX 12 kb)
